# Prediagnostic Serum Immune Marker Levels and Multiple Myeloma: A Prospective Longitudinal Study Using Samples from the Janus Serum Bank in Norway

**DOI:** 10.1158/1940-6207.CAPR-24-0501

**Published:** 2025-03-28

**Authors:** Simona Herdenberg, Carl Wibom, Esmeralda J.M. Krop, Hilde Langseth, Roel Vermeulen, Sophia Harlid, Wendy Yi-Ying Wu, Florentin Späth

**Affiliations:** 1 Department of Diagnostics and Intervention, Oncology, Umeå University, Umeå, Sweden.; 2 Department Population Health Sciences, Institute for Risk Assessment Sciences, Faculty of Veterinary Medicine, Utrecht University, Utrecht, the Netherlands.; 3 Department of Research, Cancer Registry of Norway, Norwegian Institute of Public Health, Oslo, Norway.; 4 Department of Epidemiology and Biostatistics, School of Public Health, Imperial College London, London, United Kingdom.; 5 Myeloma Research Laboratory, School of Biomedicine, Faculty of Health and Medical Sciences, University of Adelaide, Adelaide, Australia.; 6 Precision Cancer Medicine Theme, South Australian Health and Medical Research Institute (SAHMRI), Adelaide, Australia.

## Abstract

Multiple myeloma is preceded by monoclonal gammopathy of undetermined significance (MGUS). Only a minority of patients with MGUS will develop multiple myeloma, but precise prediction of progression is impossible using routine clinical biomarkers. Changes in the levels of blood immune markers can help predict disease progression. Data remain inconsistent for some markers of interest such as monocyte chemotactic protein-3 (MCP-3), macrophage inflammatory protein-1 alpha (MIP-1α), fibroblast growth factor-2 (FGF-2), vascular endothelial growth factor (VEGF), fractalkine, and transforming growth factor-alpha (TGF-α). We aimed to investigate the associations between the prediagnostic serum levels of these candidate biomarkers and future multiple myeloma risk, as well as to assess marker changes over time. We performed a nested case–control study using prospective samples from the Janus Serum Bank in Norway to investigate associations between multiple myeloma risk and prediagnostic serum levels of MCP-3, MIP-1α, FGF-2, VEGF, fractalkine, and TGF-α. The study included 293 future multiple myeloma cases with serum samples collected 20 years (median) before multiple myeloma diagnosis and 293 matched cancer-free controls. Patients with multiple myeloma had an additional prediagnostic sample collected up to 42 years before diagnosis to identify marker changes over time. Markers with >60% detection rate (MIP-1α, VEGF, and TGF-α) were included in the statistical analysis. We observed no statistically significant associations between multiple myeloma risk and serum levels of MIP-1α, VEGF, or TGF-α in samples collected 20 years before diagnosis. However, TGF-α levels decreased significantly closer to the diagnosis in patients with multiple myeloma (*P* < 0.001). The decrease in TGF-α levels may reflect subtle microenvironmental changes related to multiple myeloma progression.

**Prevention Relevance:**

This study observed a decline in TGF-α serum levels closer to multiple myeloma diagnosis, which may aid in predicting multiple myeloma progression and early detection, although validation in other longitudinal cohorts is needed.

## Introduction

More than 170,000 individuals worldwide are diagnosed with multiple myeloma each year, establishing it as one of the most prevalent hematologic malignancies (1). Multiple myeloma is preceded by monoclonal gammopathy of undetermined significance (MGUS), a frequently occurring premalignant condition observed in approximately 6% of individuals aged >60 years (2). Notably, only approximately 10% of MGUS individuals will go on and develop multiple myeloma (3). The strongest predictors of multiple myeloma progression risk utilized in clinical practice include the level and type of the monoclonal protein, free light-chain ratio, suppression of uninvolved immunglobulins, and number of monoclonal plasma cells in the bone marrow (4, 5). These established biomarkers are insufficient to accurately determine the risk of multiple myeloma progression in individual patients. As MGUS prevalence is likely to increase due to population aging, leading to a significant burden for the community and healthcare systems (6, 7), there is a need for novel blood markers to predict the onset of multiple myeloma (8).

The originating clonal cells present in MGUS may emerge decades before the onset of overt multiple myeloma, with early genetic events that often arise before 30 years of age (9). Given this long preclinical phase, monitoring blood immune marker levels could provide valuable insights for predicting multiple myeloma development. Using prospective samples from the European Prospective Investigation into Cancer and Nutrition study, Vermeulen and colleagues (10) observed associations between multiple myeloma risk and low blood levels of monocyte chemotactic protein-3 (MCP-3), macrophage inflammatory protein-1 alpha (MIP-1α), fibroblast growth factor-2 (FGF-2), vascular endothelial growth factor (VEGF), fractalkine, and transforming growth factor-alpha (TGF-α). Two studies evaluated these findings but produced inconsistent results (11, 12). Using prospective longitudinal blood samples from the Northern Sweden Health and Disease Study, we confirmed these previous findings. In addition, we observed a decline in the levels of MCP-3, FGF-2, VEGF, fractalkine, and TGF-α in multiple myeloma cases over time, possibly indicating multiple myeloma progression (11). In contrast, another study using prospective samples from the Prostate, Lung, Colorectal, and Ovarian Cancer Screening Trial in the United States observed no associations between multiple myeloma risk and FGF-2, VEGF, and TGF-α levels (12).

However, studies reporting on these associations were either relatively small (10, 11) or only included one sample per participant (10, 12), collected a median of 4 to 8 years before diagnosis (10–12). Therefore, these studies do not provide accurate information relating to marker–disease associations at earlier stages of multiple myeloma development and/or do not allow the study of immune marker changes during disease progression. To reliably identify associations between blood immune marker levels of MCP-3, MIP-1α, FGF-2, VEGF, fractalkine, and TGF-α and the future risk of multiple myeloma, we performed a case–control study using prospective samples from the Janus Serum Bank in Norway. The study included 293 future multiple myeloma cases and an equal number of matched cancer-free controls, with samples collected 20 years (median) before diagnosis. In addition, to identify the potential utility of blood immune marker changes in the prediction of multiple myeloma, we selected multiple myeloma cases with an additional prediagnostic blood sample drawn as early as 42 years before myeloma diagnosis.

## Materials and Methods

### Study cohort and design

This observational study followed the STROBE-ME guidelines (13) to ensure comprehensive reporting. We conducted a longitudinal nested case–control study using prospective blood samples from the Janus Serum Bank in Norway (14, 15). Briefly, the Janus Serum Bank includes health survey and blood donor samples collected from 318,628 individuals between 1972 and 2004. All samples are maintained at a temperature of −25°C. Through linkage to the Cancer Registry of Norway (16), we identified multiple myeloma cases with two (or more) prediagnostic serum samples per participant (*n* = 293). In multiple myeloma cases, we selected the first prediagnostic serum sample that most closely preceded the myeloma diagnosis (first sample) and one additional sample that most closely preceded the first sample (additional sample). The first and additional samples were collected at a median of 20 and 26 years before the multiple myeloma diagnosis, respectively. The cases had no other cancer diagnosis prior to multiple myeloma diagnosis, except for nonmelanoma skin cancer. All controls (*n* = 293) had a single blood sample per individual available and were individually matched to cases’ first samples on sex, age (± 6 months), date of blood draw (corresponding to the first sample in cases, ±3 months), and type of sample collection (population-based cohort or blood donor samples).

Eligible controls were individuals who were free of cancer (except for nonmelanoma skin cancer) at the date of incident case’s myeloma diagnosis. We estimated that a sample size of approximately 600 participants (293 cases and 293 matched controls) would provide more than 80% power to detect a small case–control difference in immune marker levels (Cohen’s *d* = 0.2), assuming a two-sided α of 0.05. In this study, all blood samples underwent the same sampling preprocessing procedures, except for 27 samples that were lyophilized, and iodoacetate was added to two samples before storage. Information on body mass index (BMI) and smoking was obtained from survey data at the Norwegian Institute of Public Health. Ethical approval for this study was granted by the Regional Committees for Medical and Health Research Ethics in Oslo, Norway (reference numbers 2017/2192 and 9876).

### Laboratory analyses

Using 100 µL of serum, immune marker levels were quantified in duplicate using Luminex multiplex assays (Merck Millipore) for all samples (*n* = 879). To minimize the potential influence of the analysis plate on marker–disease associations and immune marker trajectories, paired case–control sets, along with additional samples from cases, were grouped together on the same analysis plate but in a randomized sequence. Laboratory personnel were blinded regarding sample identity and performed all analyses according to the manufacturer’s protocol. The manufacturer’s standard curve range for the assay was 3.2 to 5,000 pg/mL in all markers. Marker concentrations within this range were reported directly. For values below the lower limit of the standard curve but within the extrapolated range, concentrations were estimated and reported by the Luminex system. The distribution of measurements across the standard curve, extrapolated range, and out-of-range (OOR) categories for each marker is detailed in Supplementary Table S1. Serum immune marker levels were detectable (including values reported within the standard curve and the extrapolated standard curve) in 32.1% (MCP-3), 65.3% (MIP-1α), 50.9% (FGF-2), 72.1% (VEGF), 34.9% (fractalkine), and 82.2% (TGF-α) of all analyzed samples. Derived from duplicate immune marker measurements, intra-assay coefficients of variation were in mean 10.4% (MCP-3), 11.9% (MIP-1α), 13.9% (FGF-2), 12.4% (VEGF), 13.2% (fractalkine), and 11.7% (TGF-α).

### Statistical analyses

Values outside the standard curve—either below the extrapolated range (<OOR) or above the upper limit (>OOR)—were categorized as missing as no numerical values were provided for these measurements. Immune marker measurements with coefficients of variation values ≥40% were deemed unreliable and categorized as missing to maintain data reliability. Markers were classified as detectable if their concentrations fell within the standard curve or the extrapolated range, whereas OOR values and unreliable measurements were treated as missing. Immune markers with detectable concentrations (i.e., concentrations within the standard curve or the extrapolated range) in >60% of all measurements (17) were included in statistical analyses. As a result, MIP-1α, VEGF, and TGF-α were analyzed, whereas MCP-3, FGF-2, and fractalkine were omitted from further analyses.

To address missing concentration data (i.e., OOR values and unreliable results) in MIP-1α, VEGF, and TGF-α, we generated 20 imputed datasets using multiple imputation (mice package), assuming data were missing at random. The imputation model incorporated relevant covariates, including age at blood draw, sex, duration between blood draw and diagnosis, smoking status, BMI, analysis plate, and disease status (case/control; refs. 18, 19). We used log_10_ transformation to normalize the distribution of immune marker levels. Spearman’s ρ was used to evaluate the correlation between the immune markers. Violin plots were used to visualize the distribution of immune marker levels across different sample groups.

To investigate continuous immune marker levels and multiple myeloma risk (using the first blood sample in each case), we applied conditional logistic regression to compute HRs. This analysis was adjusted to account for potential confounding factors, including smoking status [recognized as a cancer risk factor that may influence immune marker levels (20)] and BMI [recognized as a multiple myeloma risk factor (21)]. In addition, we performed an analysis stratified by time, categorizing the data based on the duration from blood draw to multiple myeloma diagnosis (<8 years, 8–25 years, and >25 years).

To investigate immune marker changes over time in multiple myeloma cases and controls, we used linear mixed modeling (lme4 package) as previously described (11, 19). In these analyses, the fixed effects incorporated in the model were disease status (case/control), the interaction between disease status and the duration from blood draw to diagnosis (in controls, the duration from blood draw to the time of diagnosis of the matched case), BMI, and smoking status. Random effects were included for each individual and case set (consisting of the first and additional samples in cases and one matched control sample; refs. 11, 19). We conducted statistical analyses using version 4.2.3 of the R Project for Statistical Computing, RRID: SCR_001905. A Code Ocean capsule has been created. The capsule contains a demonstration script and simulated data to illustrate the analytical workflow. It can be accessed at https://codeocean.com/capsule/6705232/tree/v1.



### Institutional review board statement

Our study was conducted in accordance with the Declaration of Helsinki and approved by the Regional Committees for Medical and Health Research Ethics, Oslo, Norway (Regionalekomiteer for medisinsk og helsefaglig forskningsetik, no. 2017/2192/REK sør-øst and 9876).

### Consent statement

The samples in Janus Serum Bank were collected and processed on the basis of consent for use in cancer research, in accordance with the regulations at the time of collection. During the bank’s first years, the donors gave their broad consent for their samples to be used for “research.” Samples collected in 1997 and onward are based on an explicit informed consent. The Norwegian Data Protection Authority has approved the use of data and serum samples collected in the period 1972 to 2009, based on a broad consent from the donors, partly written. The donors are free to unconditionally withdraw their consent at any time, in which case their samples will be destroyed and data will be deleted. The donors are informed about the ongoing research projects at the Cancer Registry of Norway’s web pages.

### Data availability

Data are available from the Janus Serum Bank (https://www.kreftregisteret.no/en/Research/Janus-Serum-Bank/for-researchers/) upon reasonable request.

## Results

This study included 586 serum samples from 293 multiple myeloma cases (293 first samples and 293 additional samples) and 293 single serum samples from matched cancer-free controls. All multiple myeloma cases were diagnosed between 1978 and 2016. For multiple myeloma cases, the first and additional blood samples were obtained at a median of 20 and 26 years before diagnosis, respectively (Table 1; Supplementary Fig. S1).

**Table 1.** Baseline characteristics of the study subjects and blood samples.

	Cases		Controls	
	*n*	%	*n*	%
	293	100	293	100
Age, median (IQR)^a^				
Multiple myeloma diagnosis	70.2 (13.7)		—	
First blood sample	51.5 (8)		51.5 (7.4)	
Additional blood sample	45.0 (8.5)		—	
Sex^a^				
Males	159	54	159	54
Females	134	46	134	46
BMI				
<18.5	0	0	3	1
18.5–24.9	109	37	120	41
25.0–29.9	107	37	94	32
>30.0	41	14	34	12
Unknown	36	12	42	14
Smoking				
Never smoked	104	36	81	28
Former smoker	86	29	76	26
Current smoker	67	23	94	32
Unknown	36	12	42	14
Year of diagnosis				
1978–1987	11	4	—	
1988–1997	48	16	—	
1998–2007	99	34	—	
2008–2016	135	46	—	
Years prior diagnosis				
First sample, median (IQR)^a^	19.6 (13.8)		19.7 (13.9)^b^	
<8 years	41	14	41	14
8–25 years	170	58	172	58
>25 years	82	28	80	27
Years prior diagnosis				
Additional sample, median (IQR)	25.7 (12.5)		—	
<8 years	9	3	—	
8–25 years	131	45	—	
>25 years	153	52	—	

a Matching factors included age, sex, blood draw date, and type of sample collection.

b Time from blood draw to diagnosis of incident case.

There was an uneven distribution of smoking status between the groups, with a higher percentage of current smokers observed in controls (32%) than in cases (23%; *P* = 0.02, chi-square test). The other covariables were balanced between both groups (Table 1).

Statistical analyses were restricted to immune markers with >60% detection among all measurements (i.e., MIP-1α, VEGF, and TGF-α). When investigating the controls separately, we observed no substantial correlation between age or storage time and immune marker levels. The Spearman’s ρ correlations for age with MIP-1α, TGF-α, and VEGF were −0.083 (95% CI, −0.22 to 0.06), −0.0095 (95% CI, −0.14 to 0.12), and 0.066 (95% CI, −0.07 to 0.20), respectively, indicating a consistent lack of correlation between age and these markers. The Spearman’s ρ correlations between storage time and MIP-1α, TGF-α, and VEGF levels were 0.20 (95% CI, 0.05–0.33), −0.06 (95% CI, −0.19 to 0.07), and −0.09 (95% CI, −0.22 to 0.04), respectively, indicating very weak to weak correlations, suggesting minimal impact of storage time on marker levels. Spearman’s ρ indicated modest to moderate correlations among the investigated immune markers: 0.21 (95% CI, 0.10–0.31) for MIP-1α and VEGF, 0.30 (95% CI, 0.20–0.40) for VEGF and TGF-α, and 0.39 (95% CI, 0.29–0.48) for MIP-1α and TGF-α.

**Figure 1.**
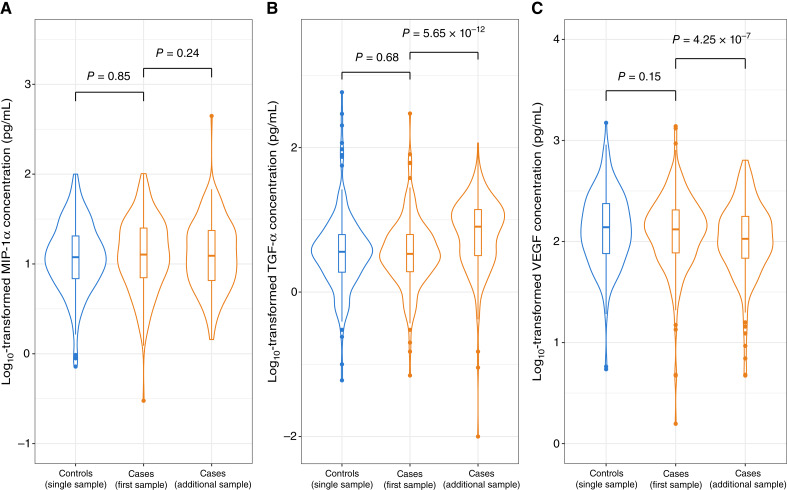
Immune marker concentrations in controls and cases. Panels display concentrations of (**A**) MIP-1α, (**B**) TGF-α, and (**C**) VEGF. Controls are shown in blue, whereas cases, including first and additional samples, are shown in orange. Statistical comparisons were performed using the Wilcoxon signed-rank test.

Median immune marker levels were similar in cases (based on the first sample collected a median of 20 years before diagnosis) compared with matched cancer-free controls (Fig. 1). For multiple myeloma cases, there was a difference in the median TGF-α levels between the first sample collected a median of 20 years before diagnosis and the additional sample collected a median of 26 years before diagnosis (*P* = 5.65 × 10^−12^). Likewise, there was a difference in median VEGF levels between the first and additional samples (*P* = 4.25 × 10^−7^). We observed no difference in the median levels of MIP-1α in multiple myeloma cases (*P* = 0.24; Fig. 1).

Associations between multiple myeloma risk and immune marker levels were assessed by conditional logistic regression analyses using the first sample in cases and matched samples in cancer-free controls. In these analyses, we observed no associations between the risk of multiple myeloma and serum levels of MIP-1α, VEGF, and TGF-α (Table 2). Including smoking status and BMI in these models, the risk estimates changed only minimally for all markers (Table 2).

**Table 2.** Conditional logistic regression with HRs and 95% confidence intervals. aHR indicates adjusted HRs for smoking status and BMI.

Marker	HR	95% CI	*P* value	aHR	95% CI	*P* value
MIP1-α	1.0	0.6–1.7	0.92	1.1	0.6–1.7	0.86
TGF-α	0.9	0.6–1.2	0.39	0.9	0.6–1.3	0.55
VEGF	0.8	0.5–1.4	0.51	0.9	0.5–1.5	0.55

Abbreviations: 95% CI, 95% confidence interval.

In the time-stratified analysis (<8 years, 8–25 years, and >25 years), we observed suggestive but not statistically significant inverse relationships between multiple myeloma risk and TGF-α and MIP-1α marker levels in samples collected <8 years before diagnosis (Table 3).

**Table 3.** Conditional logistic regression stratified by time between blood draw and diagnosis. Analyses were not adjusted for smoking status and BMI because of the lack of influence in the main model.

	<8 years (*n* = 41)^a^			8–25 years (*n* = 170)^a^			>25 years (*n* = 82)^a^		
Marker	HR	95% CI	*P* value	HR	95% CI	*P* value	HR	95% CI	*P* value
MIP1-α	0.4	0.1–2.1	0.26	1.5	0.8–2.9	0.26	0.8	0.3–1.9	0.55
TGF-α	0.5	0.1–1.7	0.25	1.0	0.6–1.8	0.89	0.8	0.4–1.5	0.46
VEGF	1.1	0.2–6.1	0.93	0.7	0.4–1.3	0.26	1.3	0.4–4.0	0.69

Abbreviations: 95% CI, 95% confidence interval.

a Number of case–control pairs included in the analyses.

**Figure 2.**
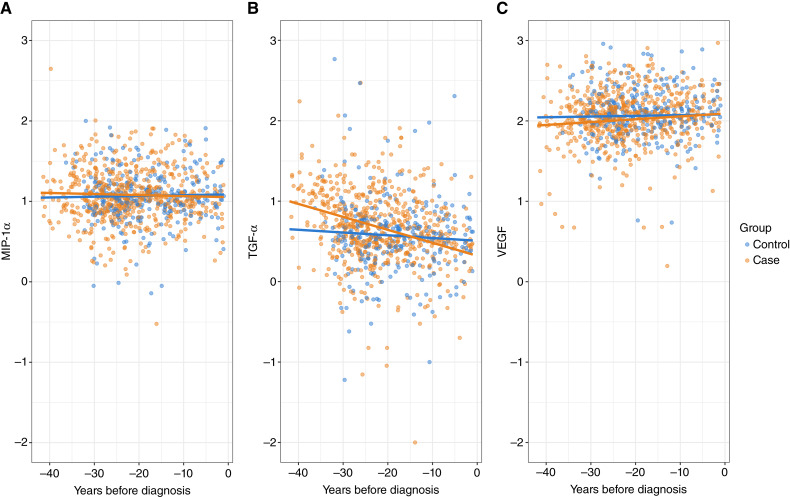
Immune marker levels over time in 293 patients with future myeloma and 293 matched cancer-free controls. Panels display levels of (**A**) MIP-1α, (**B**) TGF-α, and (**C**) VEGF. Patients with future myeloma, each with a first and an additional sample, are shown in orange, whereas matched cancer-free controls, each with a single sample per participant, are shown in blue. Lines represent linear regression across all data points.

We evaluated changes in immune marker levels over time using linear mixed effects modeling. In multiple myeloma cases, TGF-α serum levels decreased significantly closer to the diagnosis, whereas levels of VEGF and MIP-1α did not change significantly (Fig. 2; Table 4). In cancer-free controls, the serum levels of MIP-1α, VEGF, and TGF-α remained similar over time (Fig. 2; Table 4), with analyses restricted to a single blood sample per participant. In addition, we evaluated the possible influence of a tumor-induced effect on the degree of TGF-α decrease over time. To this end, we excluded all blood samples collected within 3 years before myeloma diagnosis (*n* = 31). In this analysis, the decrease in TGF-α levels remained similar (β = −0.017) compared with the analysis including all samples (β = −0.016), suggesting no strong tumor-induced effect on the observed TGF-α trajectory.

**Table 4.** Linear mixed-effects modeling and β estimates for MIP-1α, TGF-α, and VEGF.

Marker	Characteristic	β	95% CI	*P* value
MIP1-α	Intercept (controls at diagnosis)^a^	1.087	0.951 to 1.223	<0.001
	Cases compared with controls at diagnosis^a^	−0.035	−0.189 to 0.119	0.656
	Marker change over time (controls)	0.001	−0.005 to 0.007	0.751
	Marker change over time (cases)	−0.001	−0.006 to 0.004	0.596
TGF-α	Intercept (controls at diagnosis)^a^	0.508	0.331 to 0.684	<0.001
	Cases compared with controls at diagnosis^a^	−0.187	−0.383 to 0.009	0.062
	Marker change over time (controls)	−0.003	−0.011 to 0.004	0.383
	Marker change over time (cases)	−0.016	−0.021 to −0.011	<0.001
VEGF	Intercept (Controls at diagnosis)^a^	2.081	1.947 to 2.215	<0.001
	Cases compared with controls at diagnosis^a^	0.011	−0.138 to 0.161	0.881
	Marker change over time (controls)	0.001	−0.005 to 0.006	0.750
	Marker change over time (cases)	0.004	0.000 to 0.008	0.093

Abbreviations: 95% CI, 95% confidence interval.

a Diagnosis refers to the time of diagnosis of the incident case.

Sensitivity analyses excluding 29 samples with different handling (lyophilized or iodoacetate added) had no impact on the results. Similarly, applying winsorization at the 2.5th and 97.5th percentiles to minimize the influence of extreme values did not alter the findings (Supplementary Fig. S2). Furthermore, the results remained similar when the analyses were restricted to participants with complete immune marker data (i.e., excluding imputed concentration data; Supplementary Fig. S3).

## Discussion

The biomarkers currently used to monitor progression from MGUS to multiple myeloma largely reflect the tumor burden and fail to capture early changes in the microenvironment that could be assessed by changes in blood immune marker levels (22, 23). In this study, we used prospective longitudinal samples from the Janus Serum Bank in Norway to follow changes in blood immune markers preceding and/or accompanying multiple myeloma progression.

The results of our study suggest no associations between the risk of multiple myeloma and MIP-1α, VEGF, and TGF-α serum levels in samples obtained 20 years (median) before the diagnosis of multiple myeloma. Therefore, MIP-1α, VEGF, and TGF-α are not indicative of early multiple myeloma development. Notably, in multiple myeloma cases, we observed decreasing TGF-α levels closer to the diagnosis, confirming our previous findings (11) in a large and independent cohort. These observations suggest that a decrease in the blood levels of TGF-α could reflect subtle extrinsic microenvironmental changes related to multiple myeloma progression (24). Logistic regression analyses stratified by time to diagnosis indicated suggestive, but not statistically significant, inverse relationships between multiple myeloma risk and TGF-α and MIP-1α levels in samples collected <8 years before diagnosis (Table 3). However, we did not find a strong tumor-induced effect on the observed TGF-α trajectory, as corroborated by the analysis excluding all blood samples obtained within 3 years of multiple myeloma diagnosis.

It is important to recognize that previous studies reporting on the associations between multiple myeloma risk and immune markers have used substantially shorter median times between blood draw and multiple myeloma diagnosis than our study [6 years (10) and 4 years (11)]. Using prospective samples obtained 8 years (median) before multiple myeloma diagnosis, Hofmann and colleagues (12) did not observe associations between multiple myeloma risk and serum levels of FGF-2, VEGF, and TGF-α. That study included one prediagnostic sample per participant for multiple myeloma cases and MGUS individuals who did not progress. Similar to our study, Hofmann and colleagues’s (12) study was limited by a low percentage of detection of FGF-2 (32%), VEGF (68%), and TGF-α (51%). Differences in lag times between blood draw and diagnosis, as well as variations in sample types (serum vs. plasma), may contribute to the observed inconsistencies in marker–disease associations.

TGF-α functions as both a transmembrane protein and a growth factor. Upon proteolytic cleavage, it undergoes conversion to its soluble form, which then binds to the EGFR, initiating EGFR-mediated signaling pathways and promoting cell proliferation (25). TGF-α is released by various cells, including malignant, normal, and hematopoietic cells (26). Additionally, *TGFA* overexpression has been linked to malignancies such as breast cancer (27) and multiple myeloma (28). Importantly, TGF-α plays a crucial role in tumor development by driving oncogenesis and collaborating with oncogenes to promote malignant progression (29). The biological mechanisms that could promote a decrease in blood levels of TGF-α during multiple myeloma progression are unclear. Contributing factors may include discrepancies in the regulatory mechanisms between the bone marrow and peripheral blood microenvironment, which may influence cytokine and growth factor levels, acknowledging the complexity of these processes (30). In this context, a small study (*n* = 40) by Kara and colleagues (31) observed that high bone marrow TGF-α levels were significantly associated with advanced multiple myeloma stages. Interestingly, and in line with our data, whereas blood TGF-α levels were more than three times lower in patients with multiple myeloma (1.4 pg/mL) than in healthy controls (5.4 pg/mL), bone marrow TGF-α levels were higher in patients with multiple myeloma (44.7 pg/mL) than in healthy controls (28.9 mg/mL), although these findings did not achieve formal statistical significance (31). It could be speculated that differences between bone marrow and peripheral blood TGF-α levels are due to the localized production of TGF-α within the bone marrow microenvironment, leading to higher levels in this compartment compared with the systemic circulation. To evaluate this further, follow-up studies using paired bone marrow and peripheral blood samples, such as those available in the Swedish U-CAN biobank (32), are needed.

Although our study lacks longitudinal serum samples in the control group, a previous study (33) assessed the long-term stability of immune markers in cancer-free individuals and found fair to good intraindividual reproducibility for MIP-1α [intraclass correlation coefficient (ICC) = 0.57], VEGF (ICC = 0.55), and TGF-α (ICC = 0.48). Whereas the ICC for TGF-α indicated only fair reproducibility, the study observed consistent median TGF-α levels (3.9 pg/mL) in blood samples collected a median of 9.4 years apart, suggesting high temporal stability at the group level (33). The lack of repeated samples in controls prevents direct assessment of individual marker variability and represents an important limitation of our study. Although stable marker concentrations in controls reduce concerns about age or storage effects, these cannot be fully excluded. Unfortunately, including repeated control samples was not feasible at the time of study design. The inclusion of MGUS individuals remaining stable in their precursor condition would have been useful in our study, but the identification of such individuals is not possible due to the lack of information on MGUS in the Cancer Registry. Furthermore, sample volume restrictions precluded routine biomarker analysis and may have contributed to the large proportion of measures below the quantification limits. In addition, the storage temperature of −25°C is suboptimal for long-term storage and could potentially affect the stability and reliability of some markers over time.

To the best of our knowledge, this study represents one of the largest efforts to assess the associations between MIP-1α, VEGF, and TGF-α serum levels and multiple myeloma risk and progression using prospective longitudinal samples from the general population. Sampling procedures were the same in the vast majority (97%) of all samples, ensuring the comparison of samples and individuals with the same sample quality. The median age at myeloma diagnosis (70 years) reflects “real-world” conditions, consistent with the findings of other studies (34, 35). The observed sex distribution, with a slight male predominance (54% male, 46% female), is also in line with previously reported data (36). In addition, the accuracy and completeness of the Cancer Registry of Norway are high (16).

In conclusion, in this study, we observed no associations between the risk of multiple myeloma and MIP-1α, VEGF, and TGF-α serum levels in samples obtained 20 years (median) prior to the diagnosis of multiple myeloma. In multiple myeloma cases, TGF-α levels decreased significantly closer to the diagnosis. The decrease in TGF-α serum levels could reflect subtle extrinsic microenvironmental changes that accompany multiple myeloma development at a later stage. This finding warrants further investigation in independent cohorts. Longitudinal studies with repeated blood samples from both cases and controls, collected closer to myeloma diagnosis, such as those available in the Danish Blood Donor Study (37), could help validate these results and provide deeper insights into the temporal dynamics of TGF-α. Although TGF-α variability and overlap between patients and controls limit its utility as a standalone marker, it could potentially contribute to a multimarker panel providing a more comprehensive risk assessment. Such research approaches will also facilitate a more critical evaluation of TGF-α, including its potential clinical applications and limitations.

## Supplementary Material

Supplementary Table S1 Supplementary Table S1 shows categorization and handling of serum marker levels.

Supplementary Figure S1 Supplementary Figure S1 illustrates the timing of the pre-diagnostic blood sampling in relation to myeloma diagnosis among cases.

Supplementary Figure S2 Supplementary Figure S2 illustrates immune marker trajectories in myeloma cases (based on repeated samples) and matched cancer-free controls (based on a single sample per control). Panels display trajectories of (a) MIP-1α, (b) TGF-α, and (c) VEGF. To minimize the influence of extreme immune marker concentrations, analyses were conducted using winsorized data.

Supplementary Figure S3 Supplementary Figure S3 illustrates immune marker trajectories in myeloma cases (based on repeated samples) and matched cancer-free controls (based on a single sample per control). Panels display trajectories of (a) MIP-1α, (b) TGF-α, and (c) VEGF. To avoid potential effects of multiple imputation on observed marker trajectories, analyses were conducted using complete data only.
