# Supplementary material for: Prediagnostic Serum Immune Marker Levels and Multiple Myeloma: A Prospective Longitudinal Study Using Samples from the Janus Serum Bank in Norway
Source: Cancer Prev Res (Phila). 2025 Mar 28;18(7):383–91. doi: 10.1158/1940-6207.CAPR-24-0501 (PMC12209824; doi:10.1158/1940-6207.CAPR-24-0501)

Supplementary Figure S1. Sample collection time for cases before myeloma diagnosis. Each line represents one myeloma case. Timepoints of additional samples' collection (start points of red lines). Timepoints of first samples' collection (start points of blue lines). Timepoint of myeloma diagnosis (0).

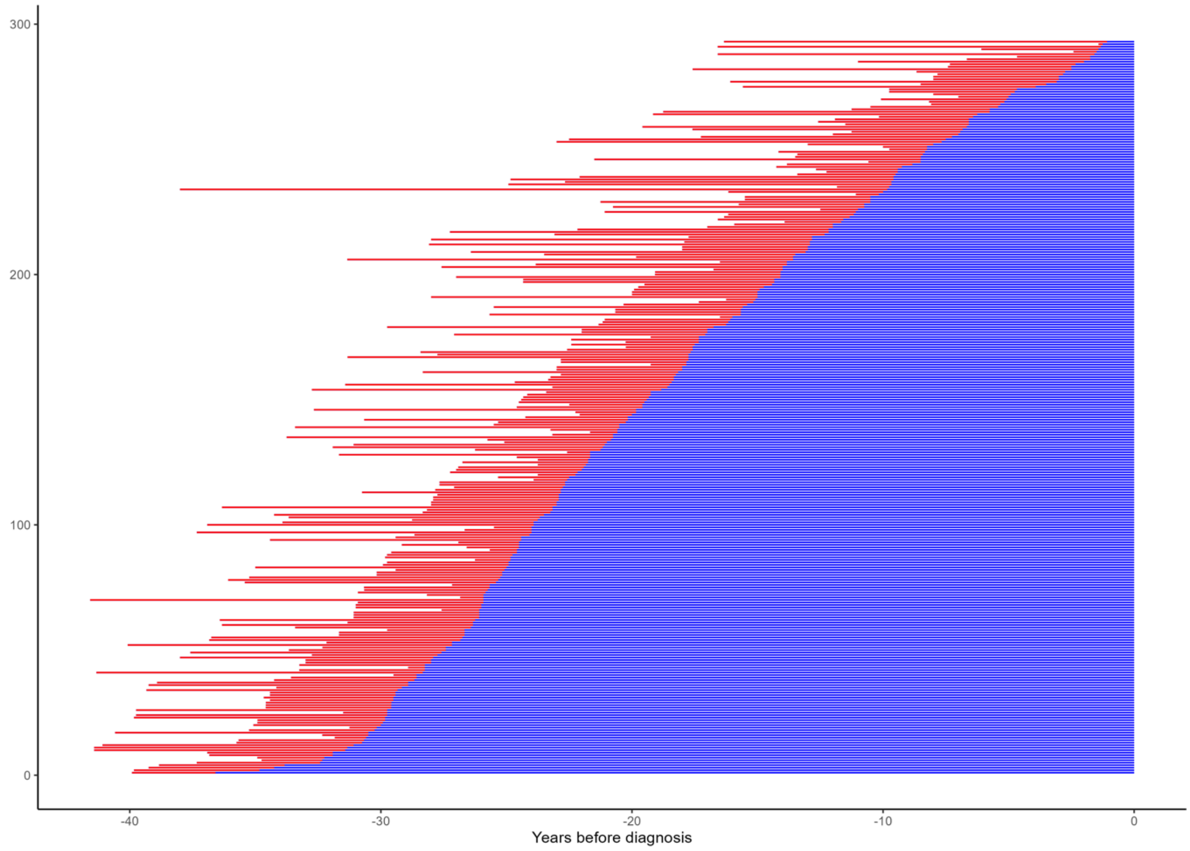

Supplement: Supplementary Figure S1 — illustrates the timing of the pre-diagnostic blood sampling in relation to myeloma diagnosis among cases. [file capr-24-0501_supplementary_figure_s1_suppsf1.pdf]
