# Supplementary material for: Prediagnostic Serum Immune Marker Levels and Multiple Myeloma: A Prospective Longitudinal Study Using Samples from the Janus Serum Bank in Norway
Source: Cancer Prev Res (Phila). 2025 Mar 28;18(7):383–91. doi: 10.1158/1940-6207.CAPR-24-0501 (PMC12209824; doi:10.1158/1940-6207.CAPR-24-0501)

Supplementary Figure S2. Immune marker levels over time in 293 future myeloma patients with a first and an additional sample available (orange), and 293 matched cancer-free controls with one sample per participant (blue) using winsorization at the 2.5th and 97.5th percentiles. Lines represent linear regression over all data points. Panels display trajectories of (a) MIP-1α, (b) TGF-α, and (c) VEGF.

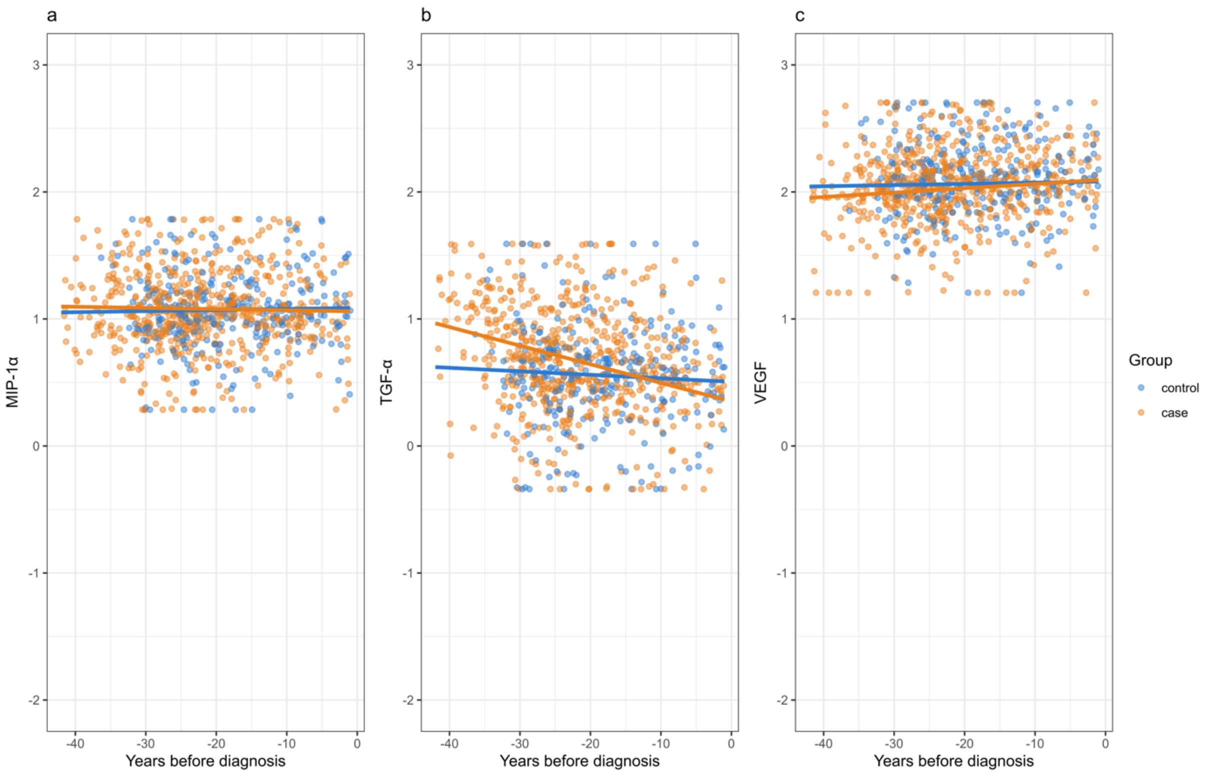

Supplement: Supplementary Figure S2 — illustrates immune marker trajectories in myeloma cases (based on repeated samples) and matched cancer-free controls (based on a single sample per control). Panels display trajectories of (a) MIP-1α, (b) TGF-α, and (c) VEGF. To minimize the influence of extreme immune marker concentrations, analyses were conducted using winsorized data. [file capr-24-0501_supplementary_figure_s2_suppsf2.pdf]
