# Supplementary material for: Prediagnostic Serum Immune Marker Levels and Multiple Myeloma: A Prospective Longitudinal Study Using Samples from the Janus Serum Bank in Norway
Source: Cancer Prev Res (Phila). 2025 Mar 28;18(7):383–91. doi: 10.1158/1940-6207.CAPR-24-0501 (PMC12209824; doi:10.1158/1940-6207.CAPR-24-0501)

Supplementary Figure S3. Immune marker levels over time in 293 future myeloma patients with a first and an additional sample available (orange), and 293 matched cancer-free controls with one sample per participant (blue). Analysis based on complete data including values within the standard curve and the extrapolated range. Lines represent linear regression over all data points. Panels display trajectories of (a) MIP-1α, (b) TGF-α, and (c) VEGF.

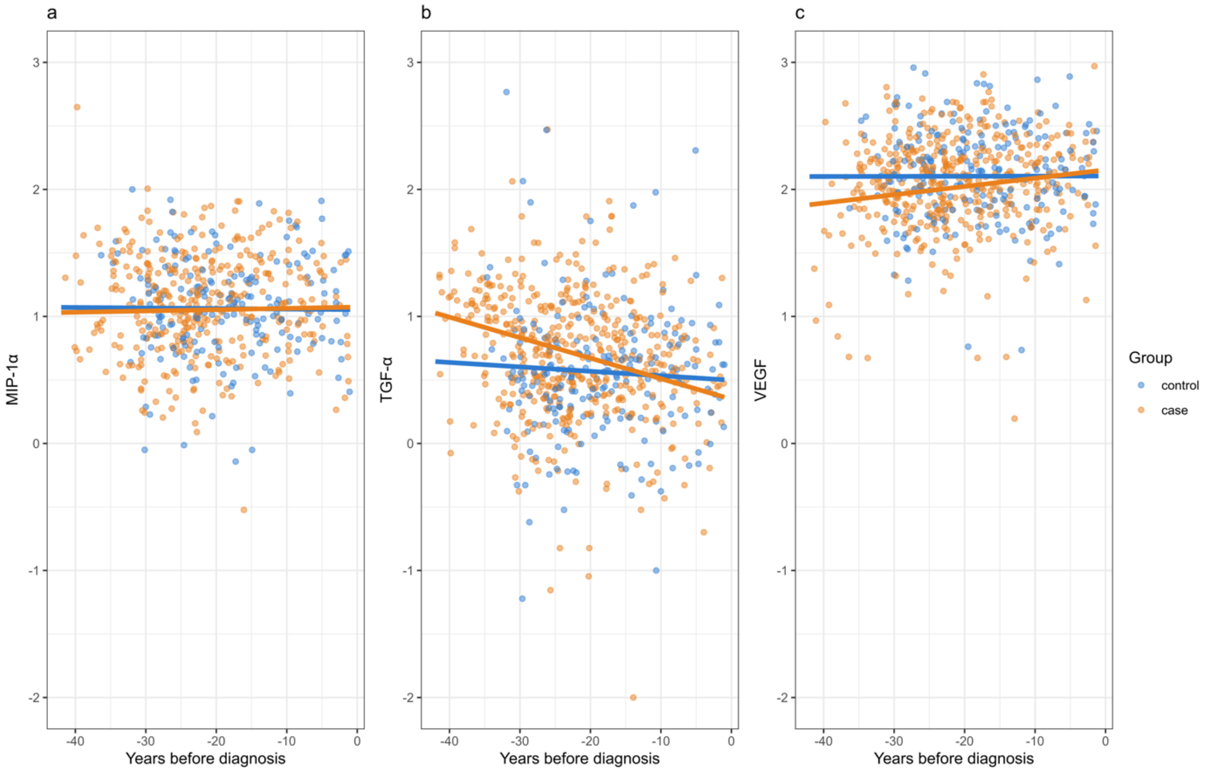

Supplement: Supplementary Figure S3 — illustrates immune marker trajectories in myeloma cases (based on repeated samples) and matched cancer-free controls (based on a single sample per control). Panels display trajectories of (a) MIP-1α, (b) TGF-α, and (c) VEGF. To avoid potential effects of multiple imputation on observed marker trajectories, analyses were conducted using complete data only. [file capr-24-0501_supplementary_figure_s3_suppsf3.pdf]
